# Hemoglobinopathies in newborns in the southern region of the Triângulo Mineiro, Brazil. Cross-sectional study: Hemoglobinopatias em recém-nascidos na região sul do Triângulo Mineiro, Brasil. Estudo transversal

**DOI:** 10.1590/1516-3180.2015.00042302

**Published:** 2015-08-21

**Authors:** Aline Menezes Carlos, Renata Andréia Volpe Souza, Bruna Maria Bereta de Souza, Gilberto de Araujo Pereira, Sebastião Tostes, Paulo Roberto Juliano Martins, Helio Moraes-Souza

**Affiliations:** I MSc. Doctoral Student in the Health Sciences Program, Universidade Federal do Triângulo Mineiro (UFTM), Uberaba, Minas Gerais, Brazil.; II PhD. Researcher at the Health Sciences Program, Universidade Federal do Triângulo Mineiro (UFTM), Uberaba, Minas Gerais, Brazil.; III BSc. Master's Student in the Health Sciences Program, Universidade Federal do Triângulo Mineiro (UFTM), Uberaba, Minas Gerais, Brazil.; IV PhD. Adjunct Professor II, Biostatistics Section, Department of Nursing, Universidade Federal do Triângulo Mineiro (UFTM), Uberaba, Minas Gerais, Brazil.; V MD. Adjunct Professor, Forensic Medicine Section, Department of Social Medicine, Universidade Federal do Triângulo Mineiro (UFTM), Uberaba, Minas Gerais, Brazil.; VI MD. Associate Professor IV, Hematology and Hemotherapy Section, Department of Medicine, Universidade Federal do Triângulo Mineiro (UFTM), Uberaba Regional Blood Center Coordinator/Hemominas Foundation, Uberaba, Minas Gerais, Brazil.; VII MD. Full Professor of the Hematology and Hemotherapy Section, Department of Medicine, Universidade Federal do Triângulo Mineiro (UFTM), Uberaba, Minas Gerais, Brazil.

**Keywords:** Fetal blood, Hemoglobinopathies, Neonatal screening, Thalassemia, Anemia, hemolytic., Sangue fetal, Hemoglobinopatias, Triagem neonatal, Talassemia, Anemia hemolítica.

## Abstract

**CONTEXT AND OBJECTIVE:**

Hemoglobinopathies are among the commonest and most widespread genetic disorders worldwide. Their prevalence varies according to ethnic composition and/or geographical region. The aim of this study was to investigate the presence of hemoglobinopathies and their association with ethnicity among 1,004 newborns, to confirm the guideline of the Brazilian National Neonatal Screening Program.

**DESIGN AND SETTING:**

Cross-sectional study conducted in a public referral hospital in the Triângulo Mineiro region, Minas Gerais, Brazil.

**METHODS:**

Qualitative assessment of hemoglobin was performed through electrophoresis on cellulose acetate: at alkaline pH to identify the hemoglobin (Hb) profile and at acid pH to differentiate Hb S from Hb D and Hb C from Hb E and others that migrate to similar positions at alkaline pH. Neutral pH was used to detect Hb Bart's identified in alpha thalassemia (α-thal). The elution method after electrophoresis was used to quantitatively assess hemoglobins.

**RESULTS:**

There was predominance of α-thal, with 105 cases (10.46%), followed by Hb S with 61 cases (6.08%, comprising 46 Hb AS, 2 Hb SS and 13 Hb S/α-thal), 9 cases (0.9%) of Hb AC and 6 cases (0.6%) suggestive of beta thalassemia (β-thal). The frequency of hemoglobinopathies was significantly higher among Afro-descendants.

**CONCLUSIONS:**

These findings corroborated of the National Neonatal Screening Program for diagnosing sickle cell disease and Hb C, Hb D, Hb E and β-thal hemoglobinopathies.

## RESUMO

**CONTEXTO E OBJETIVO:**

As hemoglobinopatias estão entre as alterações genéticas mais comuns e mundialmente difundidas, variando conforme a composição étnica e/ou geográfica. Nosso objetivo foi verificar a incidência de hemoglobinopatias por métodos de triagem laboratorial e sua associação com a etnia em 1.004 recém-nascidos.

**TIPO DE ESTUDO E LOCAL:**

Estudo transversal; realizado em um hospital público de referência do Triângulo Mineiro, Minas Gerais, Brasil.

**MÉTODOS:**

Avaliação qualitativa das hemoglobinas foi realizada por eletroforese em acetato de celulose: em pH alcalino para identificação do perfil hemoglobínico, em pH ácido para diferenciação das Hb S da Hb D e Hb C da Hb E, e outras que migrem em posições semelhantes em pH alcalino e em pH neutro para detecção de Hb Bart's, identificada na talassemia alfa (α-thal); e avaliação quantitativa das hemoglobinas pelo método de eluição após eletroforese.

**RESULTADOS:**

Houve predomínio da α-thal, com 105 (10,46%) casos, seguida da Hb S, com 61 casos (6,08% - 46 Hb AS, 2 Hb SS e 13 Hb S/α-thal), 9 casos (0,9%) de Hb AC e 6 (0,6%) sugestivos de beta-talassemia (β-thal). A incidência de hemoglobinopatias foi significantemente maior em afrodescendentes.

**CONCLUSÕES:**

Esses achados reforçam a importância do Programa Nacional de Triagem Neonatal para o diagnóstico das síndromes falciformes e hemoglobinopatias C, D, E e β-thal e robustecem os dados da literatura sobre a necessidade da pesquisa da α-thal em sangue de cordão (não identificada pelo Programa Nacional) e em pacientes com anemia microcítica não responsivos à ferroterapia.

## INTRODUCTION

Hemoglobinopathies are among the commonest genetic disorders throughout the world. They include a complex group of inherited forms of anemia that are correlated with significant morbidity, and manifest at different levels of severity from barely perceptible to lethal. They are caused by mutations that affect genes that coordinate the synthesis of the globin chains of hemoglobin (Hb), thereby resulting in absence or reduced synthesis (thalassemia and hereditary persistence of fetal hemoglobin) or structural changes (sickle cell disease, Hb C, Hb D and Hb E, among others).^1^ ^2^

Sickle cell disease and thalassemia are among the commonest hemoglobinopathies. These diseases may occur in isolation or together and cause a wide range of disorders of varying severity. There are five main categories of disorders associated with severe phenotypes, for which diagnosis during pregnancy or soon after birth and genetic counseling is usually indicated. These include thalassemia major (co-inheritance of two mutations of the beta chain); sickle cell syndromes (Hb S, Hb SS, Hb SC, Hb S/thalassemia, Hb SD, Hb SO^Arab^, Hb Lepore and Hb SE); Hb E/thalassemia (co-inheritance of mutations for thalassemia with Hb E); Hb H disease; and Hb Bart's hydrops fetalis [homozygous for alpha thalassemia (α-thal) genotype --/--].^1^ ^3^

The prevalence of hemoglobinopathies varies according to the region and the ethnic composition of the population. In some countries in Europe, Africa and Asia, where there is high incidence of hemoglobinopathies, it is a public health concern, considering that thousands of children are born with these genetic disorders annually. About 50 to 80% of children with sickle cell disease and 50,000 to 100,000 children with beta thalassemia (β-thal) major die every year.^4^ ^5^ ^and^ 6

In Brazil, data on the "Guthrie test" from the National Newborn Screening Program, which was introduced in 2001, indicate frequencies of 0.03% for Hb SS, 3.7% for Hb AS, 0.02% for Hb SC, 0.0003% for Hb SD and 0.005% for Hb S/β-thal. The highest prevalence in Bahia, followed by Rio de Janeiro, Minas Gerais, Maranhão, Pernambuco and Goiás.^7^ These data, similar to those published by the Research Center for Diagnostic Support in Minas Gerais,^8^ show the limitations of high performance liquid chromatography (HPLC), the screening method currently used in this country, for identifying patients with one or two genes for α-thal and β-thal. This is due to the high proportion of Hb F (α_2_γ_2_) at birth, which during the child's development will be substituted by Hb A (α_2_β_2_) and A_2_ (α_2_δ_2_). The screening focuses on diagnosing sickle cell diseases, Hb D, Hb E, β-thal (intermediate and major) and Hb H disease. Therefore, the minor and asymptomatic forms, which are the most prevalent presentations of α-thal and β-thal, are not usually diagnosed.

With specific regard to thalassemia, some studies have indicated prevalence rates between 0.5% and 6.6% in the northern region of Brazil,^9^ between 2.5% and 19.7% in the northeast,^10^ between 0.7% and 6.6% in the central-western region,^11^ between 0.7% and 9.48% in the southeast^12^ and between 0.2% and 4.44% in the south.^13^

Detection of carriers of these genetic changes is extremely important for public health, since all levels of the healthcare system are involved due to the high prevalence and the chronic nature of the resulting diseases, with wide-ranging clinical variations.^4^ Data on the incidence and epidemiology of hemoglobinopathies have been published for several Brazilian regions. However, apart from newborn screening and with the limitations already highlighted, no study has been conducted to report these figures in the Triângulo Mineiro region.

## OBJECTIVE

This study aimed to investigate the prevalence of hemoglobinopathies in newborns, along with any associations with ethnicity, at a hospital in the southern region of the Triângulo Mineiro region, Minas Gerais, Brazil.

## METHODS

This was a cross-sectional observational study using a quantitative approach, conducted among babies born in one public university hospital. The study was approved by the institution's Research Ethics Committee (#1836/2010). 

Among the 1,330 live births between September 2011 and January 2013, those for whom written consent was given were included in this study. Umbilical cord blood samples were collected in a tube containing 1.5 mg/ml of ethylenediamine tetraacetic acid (EDTA) and were sent to a laboratory for processing. Newborns with insufficient blood for testing were excluded and thus only 1,004 newborns were eligible for the study.

Sociodemographic and gestational data on all the newborns were gathered and documented on paper and in a database. The data gathered included name, parent's telephone number, place of origin, family history of hemoglobinopathies, gender, ethnic origin and test results.

The ethnic backgrounds of each participant and their ancestors were evaluated. In relation to the participants, ethnicity was subjectively characterized according to race, as established by the Brazilian Institute for Geography and Statistics (IBGE).^15^ The ethnicity of ancestors was ascertained by asking the participants and/or their guardians about the ethnic origin of the previous two generations (parents and grandparents) of the participant's family. Thus, three ethnic groups were established: African descendants, European descendants and unknown ancestry.

The investigation of hemoglobinopathies used the following laboratory tests: Hb electrophoresis on cellulose acetate at pH 8.6 to identify abnormal hemoglobin profiles; electrophoresis on agar phosphate at pH 6.2 to differentiate between Hb S and Hb D, Hb C and Hb E and others that migrate to a similar position at pH 8.6; electrophoresis on cellulose acetate at neutral pH to detect Hb H and Hb Bart's suggestive of α-thal; quantification of Hb A, Hb Fetal and Hb A_2_; and cytological investigation of Hb H inclusion bodies and Heinz bodies after vital staining with 1% brilliant cresyl blue to investigate suspected α-thal and unstable Hb.^16^

The newborns were divided into two groups to analyze the hematological parameters of the patients with hemoglobinopathies. The first, named the Normal Group, was composed of newborns with no hemoglobinopathies; and the second, the Case Group, had at least one type of abnormal hemoglobin.

Descriptive analysis, including absolute frequencies and percentages, means and standard deviations was used. The chi-square (χ^2)^ test was used to identify significant associations between categorical variables and the Student t test was used to compare the means of hematological variables between the Normal and Case Groups. The significance level was set at 5%. The hemoglobinopathy incidence rate was calculated by dividing the total number of live births in the hospital by the number of newborns identified with a specific hemoglobinopathy over the course of the study period.

## RESULTS

Out of the 1,004 samples analyzed, 50.1% were from females and 49.9% from males. The electrophoresis results were altered in 16.73% (n = 168) of the cases, i.e. abnormal hemoglobins were identified in one in every six newborns (1:6).

The most frequent hemoglobinopathy was α-thal (n = 105), i.e. one newborn in every 9.6 live births (1:9.6), and 13 of these cases were associated with Hb S (1:77.2). Sixty-one of the newborns were positive for Hb S (1:16.5); 46 were heterozygous (1:21.8), 13 (1:77.2) were associated with α-thal and two were homozygous (1:502). Additionally, nine newborns were heterozygous for Hb C (1:111.6) and six were suggestive of beta thalassemia (1:167.3) (Table 1).

There were no significant differences in abnormal hemoglobins with regard to gender or the place of origin of the newborns. However, a significantly higher proportion of Afro-descendants (16.94%) had abnormal hemoglobins (P < 0.0001) (Table 2).

**Table 1:**
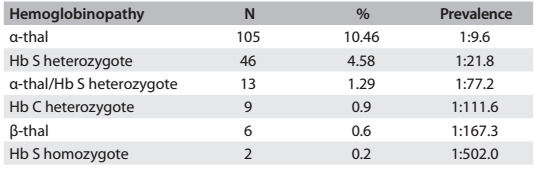
Incidence of hemoglobinopathies as observed through neonatal screening performed between 2011 and 2013 in a hospital in Brazil (n = 1,004)

**Table 2:**
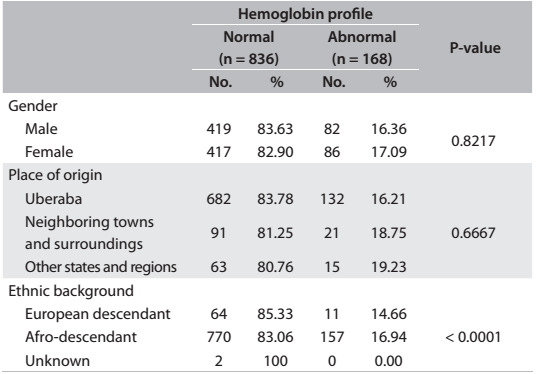
Absolute distribution and percentages of the newborns with normal and abnormal hemoglobins, stratifid according to gender, place of origin and ethnicity

## DISCUSSION

In total, around 1,160 abnormal hemoglobin values and around 300 different types of thalassemia have been described.^2^ ^17^ The process of miscegenation of the Brazilian population resulting from migration is reflected in regional characteristics and is considered to be the main factor in the distribution of abnormal genes in globin chains characteristic of the sickle cell and thalassemia syndromes. The rate of occurrence of these disorders in the Brazilian population as a whole, excluding asymptomatic carriers of α-thal, is estimated to be around 4%.^7^ ^18^

As already mentioned, the Brazilian neonatal screening program (which currently uses high-resolution liquid chromatography, HPLC) fails to identify patients with one or two abnormal genes relating to α-thal and individuals with β-thal minor due to high Hb F concentrations. This certainly explains the observed low rates of hemoglobinopathies other than Hb S, Hb C, Hb D and Hb E, as presented in official reports and even in some studies.^7^ ^18^ ^19^

The most common hemoglobinopathy in the region of Uberaba, similar to other studies conducted in Brazilian regions where there is a predominance of Afro-descendants, has been found to be α-thal (1:9.6).^10^ ^20^ ^21^ This differs from the states of Rio Grande do Sul and Paraná, where there are fewer Afro-descendants.^13^ It is known that the most common genotype of α-thal in the Brazilian population is the -α^3.7^ deletion, which is directly related to the black population.

The results from a pilot study that investigated the genotypes of 353 newborns in the same institution showed that the incidence of α-thal was 10.2%, and that 94.44% (34/36) of the diagnosed cases had the -α^3.7^ deletion and 5.56% had the -α^4.2^ deletion. Furthermore, apart from the -α^3.7^ deletion, the great majority (93.5%) of the newborns with abnormal hemoglobins were Afro-descendants, which could explain the high prevalence of the α-thal found, thereby highlighting the strong influence of this ethnicity on the composition of the population of this region.

An earlier study conducted in Uberaba among patients in the same hospital reported much lower rates of hemoglobinopathies than in the present study, especially in relation to α-thal (0.2%). This large difference can be explained by the technique that was used (electrophoresis only at alkaline pH), given that the focus of the article was Hb S disease.^22^ This reinforces the need for an association of different tests in order to detect different hemoglobinopathies, especially electrophoresis at neutral pH and cytological investigation of Hb H inclusion bodies using brilliant cresyl blue, or even molecular testing. Correlation of the results from the present study with those from several other studies on Brazilian populations (rates of between 10 and 20%)^23^ ^24^ shows the reliability of the technique used here.

In Brazil, the occurrences of β-thal are the result of extensive miscegenation of the Brazilian population with large numbers of Mediterranean immigrants, estimated at about five million, from Europe and Middle East in the nineteenth and twentieth centuries, who settled mainly in the south and southeast of the country.^16^ In this study, the prevalence of β-thal (0.60%) was similar to what had previously been found in other investigations in Brazil (from 0.38% to 1%).^25^ ^26^ However, it was lower than the rate reported by Aigner et al.^23^ (5.50%) in a study carried out in Paraná, where there is a high proportion of Italian descendants.

In relation to α-thal, research using random samples from neonatal screening in the state of Minas Gerais has shown that 30% of newborns with sickle cell anemia also inherited the α-thal gene.^27^ This result is similar to those found in the states of São Paulo (23.7%),^28^ Bahia (29.1%) and Pernambuco (25.6%).^10^ ^29^ In the current study, 21.31% of the patients with Hb S also inherited the α-thal gene and therefore the frequency of α-thal in the Brazilian population is relatively high, with even higher prevalence among Afro-descendants.^20^ ^24^ ^27^

Hb S is considered to be the most prevalent hereditary red-blood cell disorder in the world. It causes severe consequences including major hematological changes. This hemoglobin variant manifests in the heterozygous form (Hb AS, sickle cell trait), in the homozygous form (Hb SS, sickle cell anemia) or in combination with other hemoglobinopathies (sickle cell disease).^7^ ^14^ In Brazil, around one in every thousand newborns is born with sickle cell disease and about 200,000 with the sickle trait, every year.^7^ Among all the newborns with sickle cell disease, around 25% do not reach the age of five if they are not diagnosed early and treated appropriately.^16^ ^18^

The frequency of Hb S observed in this study was 5.87%. Frequencies of the Hb S gene of 4.35% and 5.88% have been reported among newborns in the states of Minas Gerais and Bahia.^7^ ^18^ This is certainly related to the high prevalence of blacks in the composition of the population in the region of the present study, where only 7.5% of the mothers did not report black ancestry within their previous two generations.

Two cases of sickle cell anemia (Hb SS) were identified in this study, giving an prevalence of 1:502 newborns. In Bahia, the frequency of the SS genotype was 1:650. These frequencies vary widely according to the degree of racial admixture in the different regions of Brazil.^10^ Since the data from the municipal Health Department of Uberaba show that the prevalence of sickle cell disease in the region in 2012 was 1:1166, the high rate observed in the current study may be due to the small sample size and also to the fact that one of the infants with Hb SS was from a neighboring town (Delta). This child's parents were from northeastern Brazil, and were Afro-descendants who had migrated to work in the sugar cane plantations of the region. Similar to Hb S, Hb C is characteristic of blacks: in Africa, the frequencies range from 5 to 25%, depending on the region. In the present study, the prevalence of Hb C (0.9%) was similar to that found in other Brazilian studies, thus reinforcing the influence of African people on the formation of the local population.^6^ ^10^ ^23^ ^30^

Although no significant differences were found between the three groups separately, comparison of the prevalence of hemoglobinopathies among whites of European ancestry with the prevalence among Afro-descendants showed a highly significant difference (P < 0.0001). This again reinforces the importance of the ethnic composition of the population of this region. 18 22 27 30

Hemoglobinopathies are diagnosed through identifying abnormal hemoglobins in the laboratory. Unlike other hemoglobins, Hb A_2_ needs to be measured using analytical methods that provide greater precision, given that the diagnosis of β-thal minor depends on these values. Individuals with β-thal minor have levels that, on average, are 1-2% above the reference values for Hb A_2_.^24^

In accordance with the Brazilian Ministry of Health Protocol, all newborns diagnosed with structural hemoglobinopathies and thalassemia by means of screening tests should be reassessed after reaching six months of age, using tests to confirm the result. However, many infants are not brought back for the tests to be repeated for diagnostic confirmation, and active searches are not always successful.^7^ Moreover, the diagnostic difficulties in the post-neonatal period are well known, especially those relating to the most prevalent forms of α-thal, with deletions of only one or two genes. This is due to the absence of clinical manifestations and the mild laboratory abnormalities,^3^ ^4^ or due to diagnostic failures mainly because of technical ineptitude and low concentrations of Hb H, as is the case of the interaction of α-thal and β-thal.^24^ However, in this study, similar to others,^10^ ^20^ ^27^ ^30^ cases suggestive of α-thal were more common than were cases of heterozygous Hb S, which is a characteristic of Afro-descendants, hence demonstrating the efficiency of the methods used here.

The prevalence of hemoglobinopathies observed in this study (16.73%) is consistent with what has been reported worldwide, and with findings from other regions of Brazil, like in the southeast and northeast (between 6.84% and 18.98%).^13^ ^20^ ^24^ However, in other Brazilian studies, frequencies of hemoglobinopathies of between 0.98% and 3.54% were found.^19^ ^25^ ^31^ The possible explanations for this great variability in the frequency of hemoglobinopathies are the wide ethnic diversity of the populations studied and the different diagnostic methodologies used.^3^ ^7^ ^10^ ^20^ ^32^

Application of more than one method in the initial screening enhances the specificity and proves to be useful for detecting patients with hemoglobinopathies. The screening methods are easy to perform, inexpensive and reproducible in routine practice in laboratories. However, application of these tests separately is unreliable and not recommended for the majority of hemoglobinopathies, which highlights the need to use complementary molecular techniques, especially for α-thal in cases of association with other hemoglobinopathies.^5^ ^12^ ^16^ ^24^ ^26^

## CONCLUSION

In summary, this study shows that the frequency of hemoglobinopathies was 16.73%, and that α-thal was the most frequent of these (10.46%). We also observed that the Afro-descendant newborns had a higher proportion of abnormal hemoglobin (16.94%, P < 0.0001). Hemoglobinopathies are a public health problem in Brazil, and these findings are corroborated by data from the National Neonatal Screening Program for diagnosing sickle cell disease and Hb C, Hb D, Hb E and β-thal hemoglobinopathies.
